# Preferential evaluation of coal filling mining scheme under building pressure based on improved grey target decision-making model of game theory

**DOI:** 10.1038/s41598-024-64124-2

**Published:** 2024-07-08

**Authors:** Junpeng Ma, Chaoqun Ma, Tao Liu, Xianxiang Zhu, Song Li, Lei Jin

**Affiliations:** 1 College of Energy and Mining Engineering, Shandong University of Science and Technology, Qingdao, 266590 People’s Republic of China; 2 Shandong Energy Group Co., Jining, 272100 People’s Republic of China; 3 Xinglongzhuang Coal Mine, Yankuang Energy Group Ltd., Jining, 272102 People’s Republic of China; 4 Yangcun Coal Mine, Yankuang Energy Group Co., Jining, 272118 People’s Republic of China; 5 College of Materials Science and Engineering, Shandong University of Science and Technology, Qingdao, 266590 People’s Republic of China

**Keywords:** Environmental social sciences, Engineering

## Abstract

The problem of coal compression under buildings is common in underground mining of coal mines in China. The selection of traditional mining programme is subjective and lacks scientific rationality. In order to solve this problem, this paper studies the evaluation index system and model applicable to the selection of coal filling mining scheme under buildings. A multi-objective evaluation index system integrating economic, technical and adaptive factors is constructed. And an integrated optimization model is established, which is based on the traditional grey target model, combining the game theory optimal combination of weights with the hierarchical analysis method, entropy weight method, Critic method to determine the weights, and then introducing the TOPSIS model and the Mars distance to establish an improved grey target decision-making model. The validity of the evaluation index system and model is verified by taking the example of coal mining under pressure of buildings in five mining areas of a coal mine, which provides technical support for decision makers. This study helps to scientifically and reasonably carry out the preferred mining scheme of coal filling under building pressure.

## Introduction

The problem of coal compression under buildings is serious in the mining areas in the east-central part of China, which affects coal mining and the service life of mines^1^. Traditional mining methods are very likely to trigger the movement of overlying rock strata and surface deformation, and fill mining, as a green and efficient coal mining method, is highly effective in improving the recovery rate of coal resources and controlling surface deformation^2,3^. However, the cost investment, economic benefits and the degree of surface mining damage vary greatly among different filling schemes, which increases the complexity of the selection of filling mining schemes^4–6^. The excellent underbuilding infill mining scheme has the characteristics of high safety, low cost investment and significant economic benefits, which can not only effectively alleviate the damage of coal mining to surface buildings, but also improve the recovery rate of coal resources, and obtain the best economic benefits with the minimum construction investment and operating costs. Based on the above analysis, the scientific and reasonable selection of coal filling mining scheme under the pressure of buildings is a key issue that needs urgent attention in the central and eastern mining areas of China.

The traditional building under pressure coal mining scheme is to determine the preliminary scheme after analysing the geological mining conditions and other factors, and then through the technical and economic analysis of the preliminary scheme selection, mainly relying on human subjective experience or similar engineering analogy to determine the method^7^, this method is from the geological, technological, economic and other perspectives in order to carry out a single-objective analysis, so as to select the final mining scheme, the subjective limitations are too strong, and cannot ensure that the preferred scheme of the scientific reliability.

With the development of science and technology, scholars at home and abroad have carried out research on the evaluation index system and evaluation model for the preferential selection of coal filling mining scheme under building. In terms of evaluation index system, Nuong^8^ and other scholars established the evaluation index system of preferential selection of under-building coal mining scheme from the aspects of mining impact, economic benefits, resource extraction rate, etc. Tang^9^ and other scholars selected the mining cost, mining and cutting ratio, construction difficulty, roof safety and other indexes to establish a preferential selection of mining scheme evaluation index system, however, the current evaluation index system is not highly applicable to under-building coal mining.

In terms of evaluation models, many scholars have carried out a lot of research and proposed some theoretical models, such as mutation preference theory^10^, AHP-FUZZY theory^11^, entropy weight-CTODIM method^12^, UMT theory^13^, variable weight theory^14^, rough set theory^15^, Vague set theory^16^, etc., which provide a large number of mining programme preference Theoretical basis, but the application of these models for evaluation exists, the evaluation results are extremely vague and uncertain, and there are limitations of unclear definition boundaries and inaccurate human judgement for the preferential evaluation of coal filling mining schemes under buildings, so the above evaluation models are difficult to carry out accurate preferential evaluation of coal filling mining schemes under buildings.

Preferred coal filling mining scheme under building pressure is actually an uncertain multi-objective decision-making problem, and the grey target decision-making model can be a good solution to this kind of problem. As an important content in grey system theory, the grey target decision-making model has been widely applied in the fields of green construction evaluation^17^, combat effectiveness evaluation^18^, vehicle path optimization^19^, and coal mine safety evaluation^20–22^ in recent years. The indicator weights in the evaluation model are crucial for the accuracy of the grey target decision-making model. Considering the shortcomings of the single assignment method, such as strong subjectivity, lack of flexibility and difficulty in dealing with a large number of indicators, in order to determine the weights of indicators in a more reasonable and reliable way, scholars have carried out the research on the combination of assignment method, Liu^23^ and other scholars have used multiplicative synthesis to assign subjective–objective combinations of the AHP method and the EWM method, and Tan^24^ and others introduced the preference coefficient μ to the hierarchical analysis method and entropy weighting method to determine the subjective and objective weights for linear weighted combination, however, the simple linear weighting or multiplicative synthesis of the indicator weights is likely to cause the theoretical value of the actual value of the deviation of the situation, how to reasonably determine the evaluation of the indicator weights is the grey-targeted decision-making model is the current need to solve the problem. In addition, the traditional grey-target decision-making model also has two problems, one is the use of a single bull's-eye in the calculation of the bull's-eye distance, which can't effectively distinguish between the advantages and disadvantages of each scheme^25^, and the other is the use of the European distance in the calculation of the bull's-eye distance, which doesn't take into account the correlation between the evaluation indexes on the impact of the evaluation results^26^, and the accuracy of the evaluation results obtained is poor, and the above problems need to be further improved.

Based on the above analysis, this paper constructs a preferred evaluation index system of under-building coal filling mining scheme and an improved grey target decision-making evaluation model based on game-theoretic combination empowerment. Firstly, the preferred evaluation index system of coal filling mining scheme under the building, which integrates economy, technology and adaptability, is proposed. Secondly, the optimal combination of subjective and objective weights of hierarchical analysis method, entropy weight method and Critic method are assigned by the game theory combination assignment method^27–29^, and the integrated optimization model with the objective function of minimizing the squared deviation of the combination weights from the subjective and objective weights is constructed to obtain the optimal weights of each index. At the same time, the theory of positive and negative ideal solutions in the TOPSIS model is introduced to construct positive and negative bull's-eye, replacing the single bull's-eye in the traditional grey-targeting model, in order to avoid the problem of inaccurate ranking caused by the bull's-eye distance of multiple scenarios being the same or close to each other. Finally, the relative bull's-eye distance is calculated using the Mahalanobis distance based on the correlation coefficient matrix to consider the correlation between indicators. Using the constructed evaluation index system and evaluation model, the decision-making preference evaluation is carried out on the filling mining schemes initially selected according to geological mining conditions in five mining areas of a coal mine, with a view to obtaining scientific and efficient decision-making schemes.

## Constructing *an index* system for evaluating the preference of underpressure coal filling mining schemes

In order to carry out the preferential evaluation of the filling mining scheme, first of all, it should be clear that the purpose of using filling mining for under-construction coal mining, the purpose of filling mining is to control the surface subsidence, improve the extraction rate of coal resources, and then improve the economic benefits of coal mining, so the excellent filling mining scheme should have the advantages of two aspects at the same time: firstly, the economic benefits are excellent, and secondly, the effect of controlling the subsidence of the ground surface is good. Therefore, when evaluating the superiority of the filling mining programme, the evaluation indexes can be determined from the above two aspects.

This paper combines the characteristics of the design of coal underpressure mining scheme under the building, and follows the principles of science, accessibility and comparability in the establishment of the index system, and selects the key indicators in the selection process of the preferred coal underpressure mining scheme under the building from the aspects of economy, technology, and adaptability, the economic indicators reflect the economic benefits brought by the scheme for the enterprise, including the filling cost, the maintenance compensation, and the amount of coal extracted. The technical indicators reflect the production efficiency of the scheme in the process of implementation, including the recovery rate, coal mining efficiency and working face production capacity; the adaptability indicators reflect the impact of the scheme on the safe and stable operation of the entire coal mining system and the external geological environment^30^, including the degree of damage caused by mining, the difficulty of controlling the quality of the filling, and the degree of flexibility and adaptability. The established evaluation index system is shown in Table 1.

**Table 1:** Constructing an index system for evaluating the preference of underpressure coal filling mining schemes.

Evaluation dimension	Evaluating attributes	Typology	Abridge
Economics	Filling Costs	Quantitative (cost)	C_1_
	Maintenance reimbursement	Quantitative (cost)	C_2_
	Quantity of coal that can be extracted	Quantitative (benefit)	C_3_
Technological	recovery ratio	Quantitative (benefit)	C_4_
	Coal mining ergonomics	Quantitative (benefit)	C_5_
	Workface production capacity	Quantitative (benefit)	C_6_
Suitability	Extent of mining damage	Qualitative (cost)	C_7_
	Difficulty of filling quality control	Qualitative (cost)	C_8_
	Flexibility of adaptation	Qualitative (benefit)	C_9_

The evaluation indicators listed in the indicator layer in Table 1 are categorised into quantitative/qualitative attributes and benefit/cost attributes according to different classifications. Among them, quantitative attributes mean that the attribute data can be quantitatively calculated by specific analysis methods, while qualitative attributes mean that the attribute data rely on the subjective judgement of experts for assigning values; benefit-type attributes mean that the higher the value of the attribute, the better the system to which it belongs; while cost-type attributes mean that the lower the value of the attribute, the better the system to which it belongs. As shown in Table 1, the filling cost, maintenance compensation fee, recoverable coal volume, recovery rate, coal mining efficiency, and working face production capacity belong to quantitative attributes, and the remaining three are qualitative attributes; while recoverable coal volume, recovery rate, coal mining efficiency, working face production capacity, and flexible adaptability belong to the benefit-type indexes, and the remaining four are cost-type attributes.

## Preferential evaluation method of coal-filling mining scheme for under-construction pressure mining based on game-theoretic grey-target decision-making model

### Traditional grey-target decision-making model

The grey target theory is proposed by Professor Deng Julong of Huazhong University of Science and Technology in China for the uncertainty system of "small sample" and "poor information" as a kind of uncertainty multi-objective decision-making method, the basic principle of which is to set the area where the satisfactory effect is located as the grey target, and to take the data closest to the threshold in the sequence of a group of indexes as the standard model, and the standard model as the bull's-eye, and to rank the size of the distance from the bull's-eye by the degree of closeness between the identification scheme and the bull's-eye, so that to make the decision-making scheme's superiority and inferiority selection.

The core step in the calculation of the grey target decision model is the calculation of the bullseye distance, which is calculated as follows:

1 $$\left\{ {\begin{array}{*{20}c} {\gamma \left( {s_{i} ,s_{0} } \right) = \sqrt {\frac{1}{n}\sum\limits_{k = 1}^{n} {\gamma \left( {s_{ik} ,s_{0k} } \right)} } } \\ {\gamma \left( {s_{ik} ,s_{0k} } \right) = \left( {s_{ik} - s_{0k} } \right)^{2} } \\ \end{array} } \right.$$

where: *n* is the number of indicators; *s*_*i*_ is the ith programme to be evaluated; *s*_0_ is the ideal optimal programme, i.e., the bullseye;$\gamma (s_{i} ,s_{0} )$ denotes the grey correlation between the programme to be evaluated, *s*_*i*_, and the optimal programme, *s*_0_, i.e.,the distance from the bullseye of the programme $s_{i}$;and $\gamma (s_{ik} ,s_{0k} )$ is the bullseye coefficient for the kth indicator under the programme i. The coefficient of bullseye for the kth indicator under the programme i is also shown in the table below.bullseye coefficient of the indicator under programme i.

As can be seen from Eq. (1):

1. The traditional grey target model in the calculation of the bull's-eye distance, by taking the arithmetic average of the bull's-eye coefficients of the indicators and then open the root sign, that is, 1/n is regarded as the weight of the evaluation indicators, the method is assumed to be the same degree of influence of the evaluation indicators on the decision-making programme, but in fact, there are bound to be differences in the importance of the evaluation indicators, and therefore the evaluation results derived from the method are bound to be biased with the actual situation.
2. The traditional grey target model adopts a single bullseye for the solution of the bullseye distance and uses the Euclidean distance to calculate the bullseye distance, which does not reasonably consider the influence of the correlation between the indicators, in the actual decision-making problem, there is often a correlation between the evaluation indicators, so the influence of the correlation between the indicators should be incorporated into the consideration of the beginning of the construction of the model, in order to make the evaluation results more scientific and reasonable.

Therefore, this paper introduces the game theory-based portfolio assignment method, the positive and negative ideal solution theory based on the TOPSIS model, and the Mars distance based on the correlation coefficient matrix to improve the traditional grey-target decision-making model.

### Combinatorial empowerment approach based on game theory

After a long period of development, the indicator assignment method has been derived from three methods: subjective assignment method, objective assignment method and combined assignment method. The subjective assignment method is represented by the AHP, which is simple, flexible, and practicable, but most of them are used for quantitative analysis of qualitative problems, and the results of weighting are more subjective. The objective assignment method is represented by the EWM and the CRITIC method. The EWM is more objective and can accommodate the influence of the scoring method of some qualitative indicators, but it only relies on objective data and does not take into account the influence of the correlation between the indicators. The CRITIC method can take into account the correlation and conflict between the indicators at the same time, making up for the defects of the EWM, which only takes into account the size of the information of the indicators and does not take into account the correlation between the indicators, and the two methods are more suitable in comparison, but coal mining is a highly empirical activity, and it is still difficult to determine the optimal weights by only taking into account the influence of the objective data on the weights. Combining the AHP, EWM and CRITIC method can synthesize subjectivity and objectivity, and the results of the index weights obtained are more perfect.

In order to overcome the limitations of single assignment method, avoid the loss of information caused by a single assignment method, and improve the accuracy of weight determination. Using the game theory combination of weighting method to optimize the combination of the weights obtained by p kinds of weighting methods, comprehensively reflecting the subjectivity and objectivity of the weights of the evaluation indexes, and obtaining the optimal weights. The specific steps are as follows:

1) Construct the set of basic weight vectors.

Assuming that *p* assignment methods are used to assign weights to the evaluation indicators, the indicator weight vectors determined by each of the *p* assignment methods can be combined to form a set of basic weight vectors:

2 $$w_{i} = \left\{ {w_{i1} ,w_{i2} , \cdots ,w_{in} } \right\},\left( {i = 1,2, \cdots ,p} \right)$$

where: *n* is the number of indicators and *w*_*i*_ is the vector of indicator weights determined by the ith assignment method.

2) Construct a linear combination of weight vectors.

Any linear combination of *P* weight vectors can be expressed as:

3 $$W = \sum\limits_{i = 1}^{p} {\alpha_{i} w_{i}^{T} } ,\left( {i = 1,2, \cdots ,p} \right)$$

where: *α*_*i*_ is a linear combination coefficient and *α*_*i*_ > 0.

(3) Solving for optimal linear combination coefficients.

According to the basic principle of game theory combination assignment, with the goal of minimising the deviation between the optimal combination weights *W* and the indicator weights *w*_*k*_ determined individually by various subjective and objective assignment methods, the *p* linear combination coefficients *α* in Eq. (3) are optimally solved so as to obtain the most satisfactory combination weights W* in *W*. The optimally solved expression is as follows:

4 $$\min \parallel \sum\limits_{i = 1}^{p} {\alpha_{i} w_{i}^{T} } - w_{k}^{T} \parallel_{2} (k = 1,2, \cdots ,p)$$

where: *w*_*k*_ is the vector of indicator weights determined by the kth assignment method.

According to the matrix differentiation property, the optimized first-order derivative condition of Eq. (4) can be derived as:

5 $$\sum\limits_{i = 1}^{p} {\alpha_{i} w_{k} \cdot w_{i}^{T} = w_{k} \cdot w_{k}^{T} } (k = 1,2, \cdots ,p)$$

Its corresponding system of linear equations can be expressed as:

6 $$\left( {\begin{array}{*{20}c} {w_{1} \cdot w_{1}^{T} } & {w_{1} \cdot w_{2}^{T} } & \cdots & {w_{1} \cdot w_{p}^{T} } \\ {w_{2} \cdot w_{1}^{T} } & {w_{2} \cdot w_{2}^{T} } & \cdots & {w_{2} \cdot w_{p}^{T} } \\ \vdots & \vdots & \ddots & \vdots \\ {w_{p} \cdot w_{1}^{T} } & {w_{p} \cdot w_{2}^{T} } & \cdots & {w_{p} \cdot w_{p}^{T} } \\ \end{array} } \right)\left( {\begin{array}{*{20}c} {\alpha_{1} } \\ {\alpha_{2} } \\ \vdots \\ {\alpha_{p} } \\ \end{array} } \right) = \left( {\begin{array}{*{20}c} {w_{1} \cdot w_{1}^{T} } \\ {w_{2} \cdot w_{2}^{T} } \\ \vdots \\ {w_{p} \cdot w_{p}^{T} } \\ \end{array} } \right)$$

The linear combination coefficients $\alpha^{*} = [\alpha_{1} ,\alpha_{2} , \ldots ,\alpha_{i} ]$ calculated from Eqs. (4) to (6) are normalized as shown in Eq. (7).Thus obtain the optimal linear combination coefficients.

7 $$\alpha_{i}^{*} = \frac{{\alpha_{i} }}{{\sum\limits_{i = 1}^{p} {\alpha_{i} } }}$$

(4) Solving for game-theoretically optimal portfolio weights.

Multiply the optimal linear combination coefficients obtained from Eq. (7) with the weights of the indicators obtained by solving each single assignment method respectively. And obtain the optimal portfolio weights for the game-theoretic portfolio $W^{*} = (w_{1}^{*} ,w_{2}^{*} , \ldots ,w_{n}^{*} )$. The calculation process is described in Eq. (8).

8 $$W^{*} = \sum\limits_{i = 1}^{p} {\alpha_{i}^{*} w_{i}^{T} } (k = 1,2, \cdots ,p)$$

### Improved grey target decision-making model based on game-theoretic combinatorial empowerment

Through the previous analysis, a series of deficiencies in the traditional grey target decision-making model are improved by substituting the game theory combination empowerment method into the traditional grey target decision-making model, and at the same time, introducing the theory of positive and negative ideal solutions in the TOPSIS model to construct the positive and negative bull's eye, and finally adopting the Mahalanobis distance based on the correlation coefficient matrix for the positive and negative bull's eye distance and the relative bull's eye distance solution.

**Figure 1:**
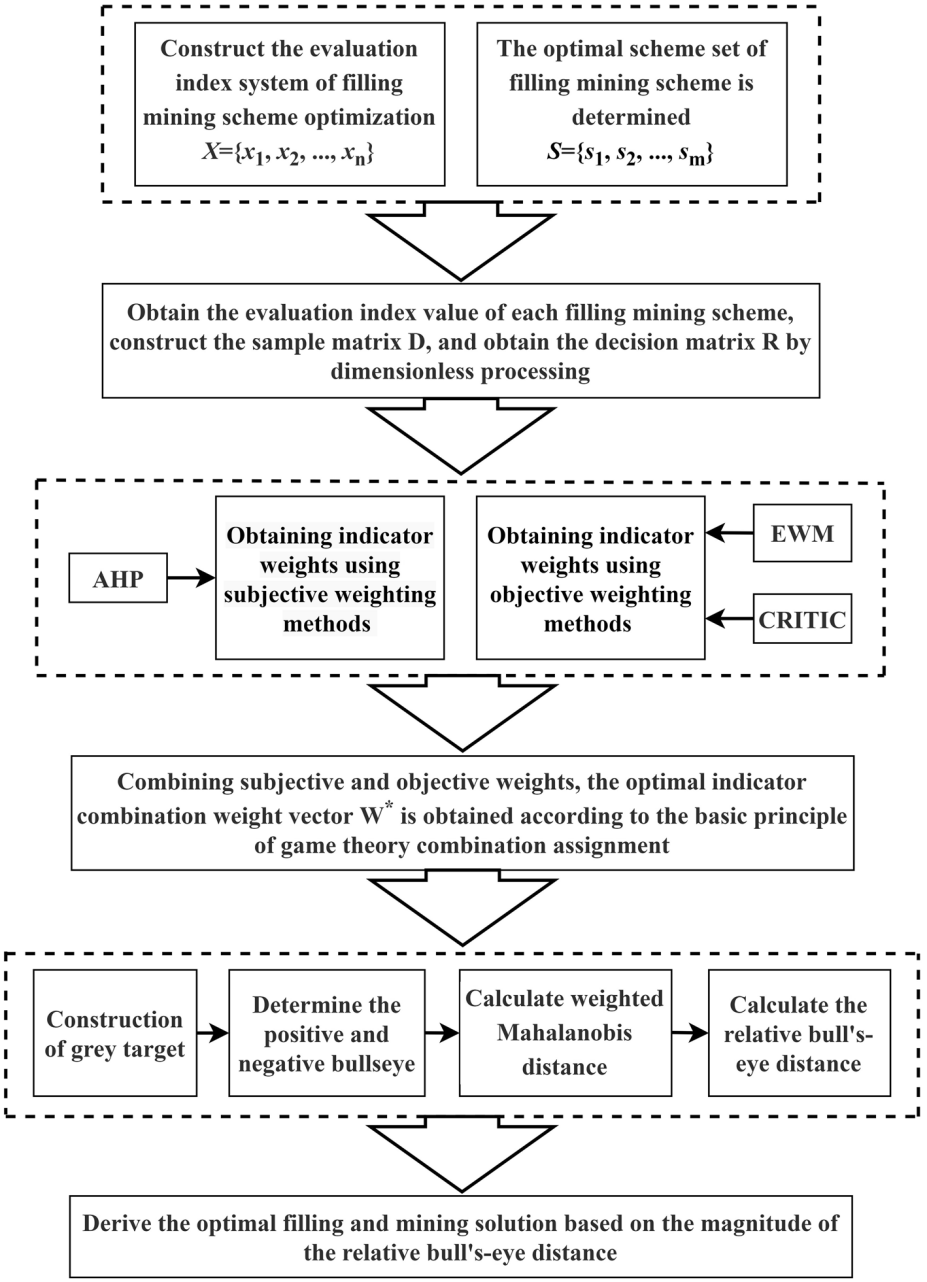
Principle and flowchart of the improved grey target decision-making model.

The principle of the improved grey target decision-making model based on game-theoretic combinatorial empowerment is shown in Fig. 1.

The specific steps are as follows:

(1) Constructing an evaluation index system for optimal selection of coal filling mining scheme under construction.

Define *n* evaluation indicators to form an indicator set: $X = \{ x_{1} , x_{2} ,..., x_{n} \}$;

(2) Determination of a preliminary Scheme for constructing an underpressure coal-fill mine.

Define *m* decision schemes to be evaluated to form a decision scheme set: $S = \{ s_{1} , s_{2} ,..., s_{m} \}$;

(3) Create sample matrix and decision matrix.

Define the sample value of the effect of the decision programme *s*_*i*_ on the evaluation indicator *x*_*j*_ to be evaluated as *x*_*ij*_(*i* = 1,2,…,*m*;*j* = 1,2,…,*n*), then the matrix of effect samples for the decision programme set *s*_*i*_ can be expressed as *D*.

Typically, the indicator attribute set $X = \{ x_{1} , x_{2} ,..., x_{n} \}$ contains three types of indicators: benefit indicators, cost indicators and interval indicators. Due to the differences in the nature and magnitude of the indicators, it is necessary to standardise the original effect sample matrix in the following steps:

Let *c*_*j*_ be the mean value of each evaluation indicator:

9 $$c_{j} = \frac{1}{m}\sum\limits_{i = 1}^{m} {x_{ij} } (i = 1,2,...,m;j = 1,2,...,n)$$

Let *r*_*ij*_ be the result of the normalisation of the indicator *x*_*ij*_.

For benefit-based indicators:

10 $$r_{ij} = \frac{{x_{ij} - c_{j} }}{{\max (\max \left\{ {x_{ij} } \right\} - c_{j} ,c_{j} - \min \left\{ {x_{ij} } \right\})}}$$

For cost-based indicators:

11 $$r_{ij} = \frac{{c_{j} - x_{ij} }}{{\max (\max \left\{ {x_{ij} } \right\} - c_{j} ,c_{j} - \min \left\{ {x_{ij} } \right\})}}$$

According to Eqs. (9) to (11) the decision matrix *R* after normalisation of the effect sample matrix *D* can be obtained and *R* can be expressed as *R*.

12 $$D = \left[ {\begin{array}{*{20}c} {x_{11} } & {x_{12} } & \ldots & {x_{1n} } \\ {x_{21} } & {x_{22} } & \ldots & {x_{2n} } \\ \vdots & \vdots & {x_{ij} } & \vdots \\ {x_{m1} } & {x_{m2} } & \ldots & {x_{mn} } \\ \end{array} } \right]\;\;R = \left[ {\begin{array}{*{20}c} {r_{11} } & {r_{12} } & \ldots & {r_{1n} } \\ {r_{21} } & {r_{22} } & \ldots & {r_{2n} } \\ \vdots & \vdots & {r_{ij} } & \vdots \\ {r_{m1} } & {r_{m2} } & \ldots & {r_{mn} } \\ \end{array} } \right]$$

Use Eq. (8) to find the game-theoretic optimal portfolio weight vector $W^{*} = \left( {w_{1}^{*} ,w_{2}^{*} , \ldots ,w_{n}^{*} } \right)$;

(4) Relative bull's-eye distance solving based on the correlation coefficient matrix of the Mahalanobis distance.

Traditional gray target model in the process of determining the bull's-eye, is to take a single ideal optimal value as the bull's-eye, when there are multiple samples obtained from the bull's-eye distance is the same or similar, the sample ranking is difficult to illustrate, so the introduction of the TOPSIS model of positive and negative ideal solution to define the positive and negative bull's-eye, when there are multiple samples of the bull's-eye distance is the same or close to the same distance, can be further judged by the distance from the worst distance from the bull's-eye distance to determine the sample ordering; Meanwhile, the traditional TOPSIS model uses the Euclidean distance to calculate the results, but the Euclidean distance only treats each variable equally and simply synthesizes the deviation of two samples on each variable, which cannot overcome the influence of correlation between attributes, while the Mahalanobis distance not only considers the correlation between attribute variables, but also takes into account the degree of difference in the values of each observation index, which can better describe the data. Similarity between the data can better describe the data to make up for the shortcomings of the Euclidean distance. In order to accurately characterize the correlation between the evaluation indicators, the correlation coefficient matrix is used to replace the covariance matrix in the Mahalanobis distance, which is introduced into the traditional gray target model, and the Mahalanobis distance based on the correlation coefficient matrix is used to calculate the positive and negative bull's-eye distances and to define the relative bull's-eye distances. The calculation process is as follows:

① Constructing positive and negative bull's-eye using the theory of positive and negative ideal solutions in the TIOSIS model. For a decision matrix *R*, if $r_{j}^{0 + } = \max \{ r_{ij} |1 < i < m\}$, then $r^{0 + } = \{ r_{1}^{0 + } ,r_{2}^{0 + } ,...,r_{n}^{0 + } \}$ is the positive bullseye of the grey-target decision; if $r_{j}^{0 - } = \min \{ r_{ij} |1 < i < m\}$, then $r^{0 - } = \{ r_{1}^{0 - } ,r_{2}^{0 - } ,...,r_{n}^{0 - } \}$ for the negative bullseye of the grey target decision, from which the weighted difference of the samples to the positive and negative bullseyes can be calculated:

13 $$u_{j} = w_{j} r_{ij} - w_{j} r_{j}^{ + } ,U = \left( {u_{1} ,u_{2} ,...,u_{n} } \right)^{T}$$

14 $$v_{j} = w_{j} r_{ij} - w_{j} r_{j}^{ - } ,V = \left( {v_{1} ,v_{2} ,...,v_{n} } \right)^{T}$$

where *U* and *V* are the weighted difference of the sample *x*_*i*_ to the positive and negative bullseye, respectively;

② The distance from the sample *x*_*i*_ to the positive and negative bull's-eye was solved separately using the correlation coefficient matrix based Mahalanobis distance, the positive and negative bullseye distances are obtained as:

15 $$\lambda_{i}^{ + } = \sqrt {U^{T} \prod^{ - 1} U}$$

16 $$\lambda_{i}^{ - } = \sqrt {V^{T} \prod^{ - 1} V}$$

where ∏ is the correlation coefficient matrix of the decision matrix *R*.

③ Eqs. (13) to (16) were used to fuse the TOPSIS model with the Mahalanobis distance to calculate the positive and negative bullseye distances for each sample. Equation (17) was used to determine the relative bullseye distance for each decision sample.

17 $$\lambda_{i}^{*} = \frac{{\lambda_{i}^{ + } }}{{\lambda_{i}^{ + } + \lambda_{i}^{ - } }}$$

The size of the relative bull's-eye distance can reflect the degree of the program's advantages and disadvantages, the smaller the relative bull's-eye distance *λ*_i_^*^ is, the higher the proximity of the sample xi relative to the positive bull's-eye, and the further the distance from the negative bull's-eye, which implies that the corresponding decision-making program is more optimal, and the decision-making program is optimal when the relative bull's-eye distance *λ*_i_^*^ reaches the minimum value.

## Application of engineering examples

### Overview of the mine area

The ground surface in the shaft field of the fifth mining area of a coal mine is flat, the ground is densely populated with villages, and there are a total of 38 buildings (structures) such as villages, schools and enterprises, etc., with the upper limit of mining elevation of -790m and the lower limit of mining elevation of -870m; the terrain is slightly higher in the northeast and lower in the southeast, with the slope of the terrain of about 0.3%; the inclination of the regional coal seams is about 4°, and the change is not big. The coal strata in the area are Carboniferous Permian Taiyuan Formation and Permian Shanxi Formation, and the main coal seams mined are the 3 Upper Coal Seam, with an average thickness of 4.56m; there are many surface buildings in the area.

### Evaluation sample construction

A coal mine five mining area belongs to the building under the legacy strip coal mining, building under the pressure of coal mining is a typical "three" mining. According to the LUFRC energy [2021] No. 242 document 《Implementing Opinions on Further Improving the Management of Coal Mining Subsidence Land and Other Work》. In principle, no new under-building mining will be carried out in the future, where under-building mining is necessary, it must be carried out by filling. Paste filling mining is favored for its advantages of reducing surface subsidence, protecting the ecological environment, making full use of gangue and other wastes, maximizing the use of resources, and mature technology, etc. Aiming at the characteristics of the importance of the surface buildings in the study area, as well as the problems of high ground stress in the case of deep mining. We choose the paste filling mining method with better effect of controlling the subsidence of the surface to mine the remaining strips under the buildings of the five mining areas of a coal mine.

In the case of paste body filling mining, the factors affecting surface subsidence can be divided into three main categories according to their nature: First, the engineering geological conditions of the mining area, the engineering geological conditions are the key natural factors affecting the effect of controlling surface subsidence in filling mining, and the overburden rock itself, the repetitive mining, and the environment of the coal body are all having an effect on the destabilization mechanism of the peripheral rock of the mining field, which then affects the surface subsidence; Second, the technical conditions of the mining, the technical conditions of the mining have a significant effect on the control of surface deformation in filling mining. Secondly, mining technology conditions, mining technology conditions have a significant impact on the control of surface deformation of filling mining, including the size of the working face, mining and filling cycle process, filling rate, and the amount of top and bottom plate migration, etc.; Thirdly, the nature of the filling body, in the process of paste filling mining, the strength of the cemented filling body is a key influence on the stability of quarry perimeter rock and the filling body, and the ratio of the filling material and the maintenance time plays a crucial role in the strength of the filling body. By improving the compressive strength and modulus of elasticity of the filler, the stability of the quarry space can be significantly enhanced. However, this initiative is also accompanied by an increase in the cost of coal mining.

For coal mining, the engineering geological conditions of the mine are natural and cannot be changed. The nature of the filling body is pre-designed for the mining conditions, while the size of the working face in the mining technical conditions is easy to change artificially. Working face dimensions include mining width, working face length and mining thickness, when designing the working face, it is necessary to ensure the stability of the coal pillar and filling body on both sides of the mining hollow area, to prevent accidents such as fall and slice of gangs, etc. For the deep paste filling mining, the influence of mining width on the effect of the control of surface subsidence is relatively slight, so this paper mainly designs the comparison scheme by changing the mining height and working face length.

Considering that the average mining thickness in this case is 4.56m, which belongs to thick coal seam mining, so the critical mining height needs to be determined firstly. The geological mining conditions of this coal mine are similar to those of the neighboring mines, and with reference to the successful experience of paste filling mining in the neighboring mines, and with reference to the actual production of other mining areas of this mine, it is determined that the critical mining height of the fifth mining area is 4.5m.In order to improve the recovery rate of coal resources and avoid the stagnation of resources caused by under-constructed coal, according to the geological and mining conditions and the characteristics of the natural geographical environment of the mine, taking into account the dual requirements of the protection of surface buildings and the guarantee of economic benefits, and combining with the successful experience of the coal mining by paste filling under the buildings of the neighbouring mines, we put forward three representative mining schemes of paste filling:

1. With a large face and long limit thickness single long wall backward fully mechanized paste filling mining method (Scheme 1), the average length of the designed working face is 210 m, and the average mining height of the designed working face is 3.5 m.
2. General face-length limited-thickness single long wall backward fully mechanized paste filling mining method (Scheme 2), with an average design working face length of 138 m and an average design working face height of 3.5 m.
3. General face long growing height single long wall backward fully mechanized paste filling mining method (Scheme 3), the average length of the designed working face is 138 m, and the average mining height of the designed working face is 4.5 m.

The above three schemes are taken as the evaluation scheme set. According to the actual engineering data calculation of the five mining areas of the mine and similar mine engineering experience, the evaluation index data, namely the index set, of each filling mining scheme is obtained, as shown in Table 2.

**Table 2:** Evaluation sample of coal filling mining plan under construction in the fifth mining area of a coal mine.

Normative layer									
Scheme	Q_1_			Q_2_			Q_3_		
	*x*_1_(yuan·t^-1^)	*x*_2_(ten thousand yuan)	*x*_3_(10,000 tons)	*x*_4_(%)	*x*_5_(t·h^-1^)	*x*_6_(t·d^-1^)	*x*_7_	*x*_8_	*x*_9_
1	317.69	23,941.75	168.5	94	20.98	2100	0.6	0.9	0.7
2	312.28	22,005.24	161.5	93	21.94	2440	0.5	0.6	0.9
3	293.42	22,468.98	190.7	95	10.59	2250	0.7	0.8	0.6

Apply the constructed evaluation index system and evaluation model to evaluate and construct sample matrix *D*. In the constructed evaluation index system for the preferred evaluation of underpressure coal filling and mining scheme, filling cost *x*_1_, maintenance compensation fee *x*_2_, mining damage degree *x*_7_, filling quality control difficulty *x*_8_ are cost-type indicators, and the rest of the indicators are benefit-type indicators. According to Eqs. (9) ~ (11), the data of each index is standardised to obtain the standardisation matrix *R*.

$$D = \left[ {\begin{array}{*{20}c} {\begin{array}{*{20}c} {\begin{array}{*{20}c} {317.69} & {23941.75} & {168.5} & {94} \\ \end{array} } & {20.98} & {2100} & {0.6} \\ \end{array} } & {0.9} & {0.7} \\ {\begin{array}{*{20}c} {\begin{array}{*{20}c} {312.28} & {22005.24} & {161.5} & {93} \\ \end{array} } & {21.94} & {2440} & {0.5} \\ \end{array} } & {0.6} & {0.9} \\ {\begin{array}{*{20}c} {\begin{array}{*{20}c} {293.42} & {22468.98} & {190.7} & {95} \\ \end{array} } & {10.59} & {2250} & {0.7} \\ \end{array} } & {0.8} & {0.6} \\ \end{array} } \right]$$

$$R = \left[ {\begin{array}{*{20}c} {\begin{array}{*{20}c} {\begin{array}{*{20}c} { - 0.688} & { - 1.000} & { - 0.283} & { - 0} \\ \end{array} .200} & {0.434} & { - 0.925} & {0.000} \\ \end{array} } & {1.000} & { - 0.200} \\ {\begin{array}{*{20}c} {\begin{array}{*{20}c} { - 0.312} & {0.704} & { - 0.717} & { - 0.800} \\ \end{array} } & {0.566} & {1.000} & { - 0.600} \\ \end{array} } & { - 0.800} & {1.000} \\ {\begin{array}{*{20}c} {\begin{array}{*{20}c} {1.000} & {0.296} & {1.000} & {1.000} \\ \end{array} } & { - 1.000} & { - 0.076} & {0.820} \\ \end{array} } & { - 0.200} & { - 0.800} \\ \end{array} } \right]$$

### Determination of optimal portfolio weights

This paper is based on the game theory combination assignment method to determine the combination weight value of each evaluation index. Firstly, three single assignment methods of AHP method, entropy weight method and Critic method are used to determine the subjective and objective weights, and the calculation steps are detailed in the literature [28, 29]. Based on the AHP method, the subjective weight is determined as *w*_1_(0.097,0.166,0.114,0.202,0.116,0.044,0.075,0.108,0.078), and based on the entropy weight method and Critic method, the objective weight is determined as *w*_2_(0.117,0.080,0.118,0.121,0.073,0.128,0.121,0.121,0.121) and *w*_3_(0.105,0.114,0.109,0.107,0.115,0.118,0.106,0.114,0.112), respectively.

**Figure 2:**
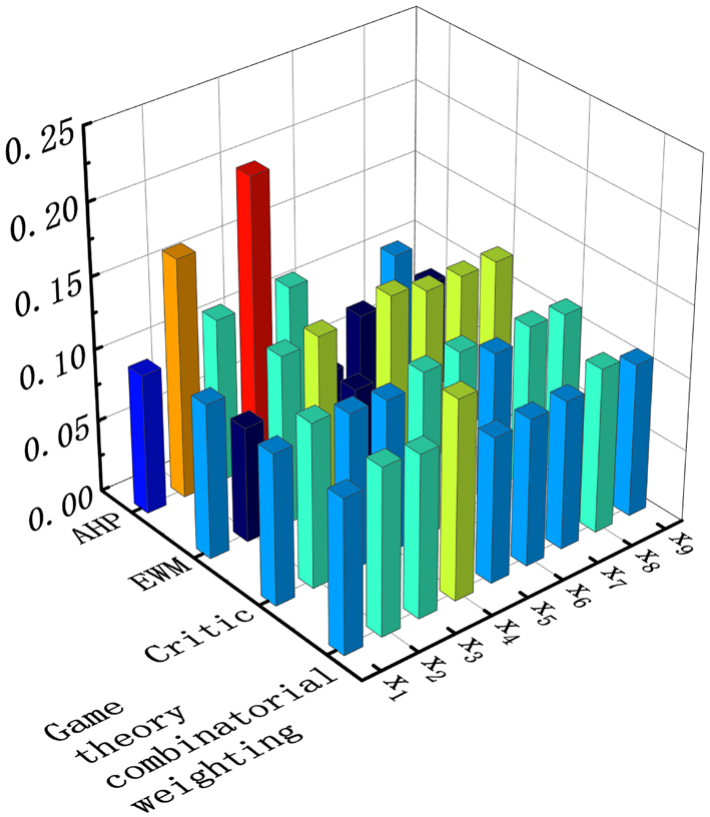
Multi-Indicator Multi-Algorithm Weights Visualisation Map.

According to Eqs.(3) to (6), the optimal linear combination of coefficients of the three sets of weight vectors is solved by using the numerical software of MATLAB to obtain $(\alpha_{1} ,\alpha_{2} ,\alpha_{3} )$ are (0.275,0.377,0.348), respectively, the three optimal linear combination coefficients obtained from the solution are substituted into Eq. (8) to obtain the optimal combination of weights W* for each indicator, and the weight vectors obtained from the calculation of the different weight determination methods are shown in Table 3. As a result, the multi-indicator and multi-algorithm weights are plotted and visualized as shown in Fig. 2. Shown.

**Table 3:** Weight vectors for different weight determination methods.

Indicator	*x*_1_	*x*_2_	*x*_3_	*x*_4_	*x*_5_	*x*_6_	*x*_7_	*x*_8_	*x*_9_
*Method*									
AHP	0.097	0.166	0.114	0.202	0.116	0.044	0.075	0.108	0.078
EWM	0.117	0.080	0.118	0.121	0.073	0.128	0.121	0.121	0.121
CRITIC	0.105	0.114	0.109	0.107	0.115	0.118	0.106	0.114	0.112
Game theory combinatorial weighting	0.107	0.116	0.113	0.138	0.101	0.102	0.103	0.114	0.106

### Improved grey-target model scheme preference

Based on the indicator normalization matrix R, positive and negative ideal solutions, i.e., positive and negative bullseyes, can be determined. Taking *r*_*i*1_ as an example, *r*_11_ = − 0.688, *r*_21_ = − 0.312, and *r*_31_ = − 1.000, where *r*_31_ has the largest normalized value of the indicator, so it is a positive bull's-eye, while *r*_11_ has the smallest normalized value of the indicator, so it is a negative bull's-eye. The resulting positive bull's-eye *r*^0+^ and negative bull's-eye *r*^0-^ of the sample are respectively:

$$\begin{aligned} r^{{0 + }} & = (1.000,0.704,1.000,1.000,0.566,1.000,0.820,1.000,1.000). \\ r^{{0 - }} & =( - 0.688, - 1.000, - 0.717, - 0.800, - 1.000, - 0.925, - 0.600, - 0.800, - 0.800). \\ \end{aligned}$$

The corresponding correlation coefficient matrix ∏ can be derived from the indicator normalization matrix *R*.

$$\prod = \left[ {\begin{array}{*{20}c} {\begin{array}{*{20}c} {\begin{array}{*{20}c} {\begin{array}{*{20}c} {1.000} & { - 0.485} & {0.896} \\ \end{array} } \\ {\begin{array}{*{20}c} { - 0.485} & {1.000} & { - 0.047} \\ \end{array} } \\ {\begin{array}{*{20}c} {0.896} & { - 0.047} & {1.000} \\ \end{array} } \\ {\begin{array}{*{20}c} { - 0.074} & {0.229} & {0.962} \\ \end{array} } \\ \end{array} } & {\begin{array}{*{20}c} { - 0.740} \\ {0.229} \\ {0.962} \\ {1.000} \\ \end{array} } & {\begin{array}{*{20}c} {0.658} \\ {0.214} \\ { - 0.886} \\ { - 0.902} \\ \end{array} } & {\begin{array}{*{20}c} { - 0.147} \\ { - 0.636} \\ { - 0.308} \\ { - 0.558} \\ \end{array} } \\ \end{array} } & {\begin{array}{*{20}c} { - 0.740} \\ {0.986} \\ {0.862} \\ {1.000} \\ \end{array} } & {\begin{array}{*{20}c} {0.024} \\ {0.229} \\ {0.422} \\ {0.655} \\ \end{array} } & {\begin{array}{*{20}c} {0.600} \\ { - 0.409} \\ { - 0.892} \\ { - 0.982} \\ \end{array} } \\ {\begin{array}{*{20}c} {\begin{array}{*{20}c} {0.658} & {0.214} & { - 0.886} \\ \end{array} } & { - 0.902} & {1.000} & {0.144} \\ \end{array} } & { - 0.902} & { - 0.263} & {0.804} \\ {\begin{array}{*{20}c} {\begin{array}{*{20}c} { - 0.147} & { - 0.636} & { - 0.308} \\ \end{array} } & { - 0.558} & {0.144} & {1.000} \\ \end{array} } & { - 0.558} & { - 0.983} & {0.704} \\ {\begin{array}{*{20}c} {\begin{array}{*{20}c} {\begin{array}{*{20}c} { - 0.740} \\ {0.024} \\ {0.600} \\ \end{array} } & {\begin{array}{*{20}c} {0.986} \\ {0.229} \\ { - 0.409} \\ \end{array} } & {\begin{array}{*{20}c} {0.862} \\ {0.422} \\ { - 0.892} \\ \end{array} } \\ \end{array} } & {\begin{array}{*{20}c} {1.000} \\ {0.655} \\ { - 0.982} \\ \end{array} } & {\begin{array}{*{20}c} { - 0.902} \\ { - 0.263} \\ {0.804} \\ \end{array} } & {\begin{array}{*{20}c} { - 0.558} \\ { - 0.983} \\ {0.704} \\ \end{array} } \\ \end{array} } & {\begin{array}{*{20}c} {1.000} \\ {0.655} \\ { - 0.982} \\ \end{array} } & {\begin{array}{*{20}c} {0.655} \\ {1.000} \\ { - 0.786} \\ \end{array} } & {\begin{array}{*{20}c} { - 0.982} \\ { - 0.786} \\ {1.000} \\ \end{array} } \\ \end{array} } \right]$$

The correlation coefficient matrix is introduced into the calculation and solution process of relative bull's-eye distance, combined with the game theory optimal combination of weights W*, and the MATLAB numerical software is used to program and solve Eqs. (13) ~ (17) to obtain the relative bull's-eye distance of each scenario *λ*_i_^*^ = (0.636,0.424,0.507). According to the relative bull's-eye distances of the three options to be selected from the model evaluation, the advantages and disadvantages of the options are ranked in the order of the relative bull's-eye distances from smallest to largest, resulting in the following ranking of the advantages and disadvantages of the options: Option 2 > Option 3 > Option 1.

### Analysis and discussion

In order to further verify the effectiveness of the improved grey target decision model in this paper, it is analyzed from the two perspectives of evaluation index weight and index correlation.

#### Analysis of indicator weights

In order to verify the role of the game theory combined assignment method introduced in this paper, from the perspective of evaluation index weights, the AHP method coupled gray target decision-making model, EWM method coupled gray target decision-making model and Critic method coupled gray target decision-making model were used to determine the relative bull's-eye distance and sequencing of each program. Compare and analyze the results of each evaluation with the relative bull's-eye distance and ranking of each option determined by the game-theoretic portfolio-empowered gray-target decision-making model.The evaluation results are shown in Table 4.

**Table 4:** Relative bullseye distance and ordering of multiple weight calculation methods coupled gray target decision-making model.

Scheme	AHP		EWM		Critic		Game theory combinatorial weighting	
	Relative bullseye distance	Sort	Relative bullseye distance	Sort	Relative bullseye distance	Sort	Relative bullseye distance	Sort
1	0.645	3	0.619	3	0.553	3	0.636	3
2	0.502	1	0.412	2	0.456	2	0.424	1
3	0.526	2	0.366	1	0.389	1	0.507	2

As can be seen from Table 4, for the same set of sample data, the evaluation results of the game theory combination of empowerment, hierarchical analysis coupled to improve the grey target decision-making model are ranked consistently, and the results are ranked as follows: Scheme 2 > Scheme 3 > Scheme 1, while the entropy weighting method, the Critic method coupled to improve the grey target decision-making model of the evaluation results of the rankings show that Scheme 3's superiority is greater than that of Scheme 2; From the numerical value, it can be seen that the relative bull's-eye distance calculation results obtained by using the hierarchical analysis method, entropy weight method and Critic method coupled to improve the grey-target decision-making model and the game theory combination of empowerment coupled to improve the grey-target decision-making model have a high degree of incoherence, and there is a situation in which the subjective weights account for a large proportion of the objective weights account for a small proportion of the objective weights, and the main reason for this is derived from the analysis: Hierarchical analysis is a subjective empowerment method used for determining the relative importance of multiple indicators. The main reason is that Hierarchical Analysis is a subjective assignment method for determining the relative importance of multiple indicators, which determines the weights of the indicators through the scoring of decision makers, involves the subjective empirical judgement of the experts, and different experts may have different views and preferences, and the evaluation results are greatly influenced by subjective factors. Entropy and Critic methods are objective assignment methods, which calculate indicator weights through quantitative data of evaluation indicators, but the determination of indicator weights lacks the support of subjective human experience, and the evaluation results lack rationality. The accuracy of the combination assignment method depends on the determination of the proportion of subjective and objective weight vectors, because coal mining is a practical activity, the accumulation of expert knowledge and experience is very important in practice, so for the selection of mining programmes, this paper determines that the subjective weight accounts for a large proportion of the objective weight as an auxiliary corrective value, which accounts for a relatively small proportion of the small. In addition, the evaluation results of the model are not only consistent with the actual results in the field, but also the degree of difference between the schemes is large, and the optimal scheme is obviously better than the other schemes, which illustrates the scientific and rationality of the game-theoretic combinatorial empowerment grey-target decision-making model.

**Figure 3:**
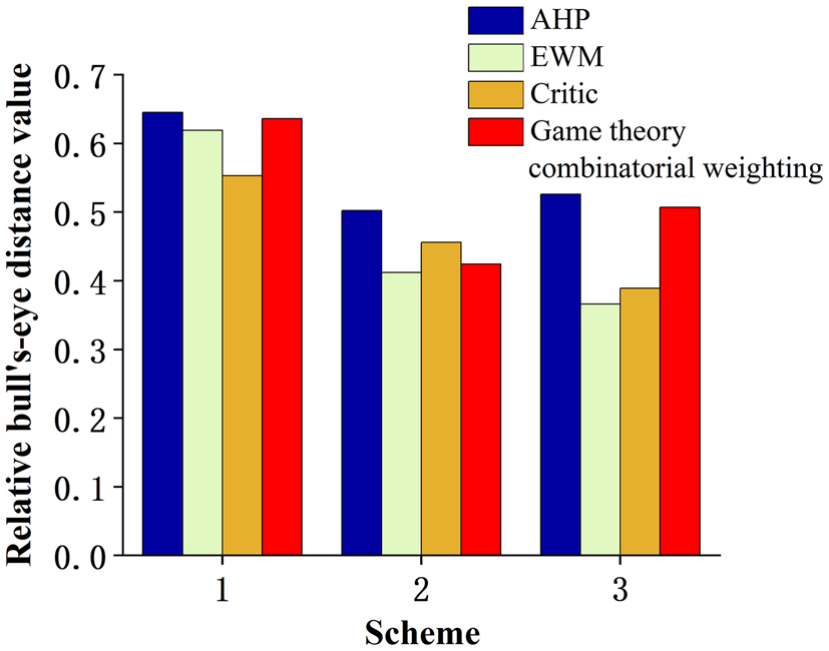
Multi-schema multi-algorithm visualisation of relative bullseye distances.

After the overall analysis, it is concluded that the optimal weights of the indicators are obtained based on the combination of game theory assignment, which takes into account the subjective experience of human beings and the objective data of practice, and is essentially a linear optimization combination of indicator weights with the goal of minimizing the deviation of the results of the individual calculation of the three weight determination methods, and achieves the coordination and unity of the results of the indicator weights of the three assignment methods. The visualisation of the relative bull's-eye distance of the multi-programme multi-algorithm is shown in Fig. 3.

#### Indicator correlation analysis

In order to verify the theory of positive and negative ideal solutions in the TOPSIS model introduced in this paper as well as the role of the Mahalanobis distance based on the matrix of correlation coefficients, the improved gray-targeted decision-making model is compared and analyzed with the traditional gray-targeted decision-making model from the point of view of whether or not to consider the correlation of the indicators.It can be seen from the data in Table 5 that the results of the sorting of Scheme 2 and Scheme 3 calculated by the two models are inconsistent, especially the center-of-target distance of Scheme 3 fluctuates a lot, and the main reason is analyzed:

1. The inconsistency of the sorting results is due to the fact that the traditional gray target decision-making model adopts the Euclidean distance for the calculation of the bull's-eye distance, ignoring the correlation between the evaluation indexes, and some of the data are double-calculated, which in turn affects the accuracy of the determination of the bull's-eye distance and the reasonableness of the program's sorting.
2. The larger fluctuation in the bull's-eye distance of Scenario 3 calculated by the two models is due to the introduction of the theory of positive and negative ideal solutions in the TOPSIS model into the traditional gray-target model, which integrally considers the distance of the samples to the positive and negative bull's-eye and further increases the differentiation of the sample bull's-eye distances, while the traditional gray-target model considers only the single bull's-eye, which results in the situation where the bull's-eye distances of the model evaluation results are close to each other.

**Table 5:** Comparative analysis of the improved gray target model and the traditional gray target model.

Scheme	Improved grey target model		Traditional grey target model	
	Relative bullseye distance	Sort	bullseye distance	Sort
1	0.636	3	0.598	3
2	0.424	1	0.426	2
3	0.507	2	0.375	1

In summary, the theory of positive and negative ideal solutions in TOPSIS model is incorporated into the traditional gray target model, and the Mahalanobis distance based on the correlation coefficient matrix is used to solve the bull's-eye distance, which reasonably takes into account the correlation between the evaluation indexes, and also takes into account the degree of difference in the values of individual indexes, and the differentiation between the relative bull's-eye distances obtained by each of the relative bull's-eye distances is higher in comparison with the Euclidean distance, which compensates for the shortcomings of the Euclidean distance, and is conducive to evaluating the degree of excellence in the sample Sorting.

**Figure 4:**
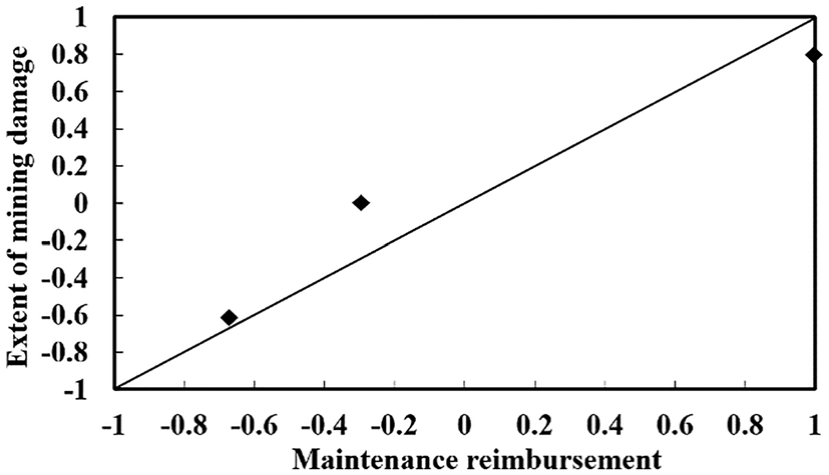
Correlation between the maintenance compensation fee and the degree of mining damage.

**Figure 5:**
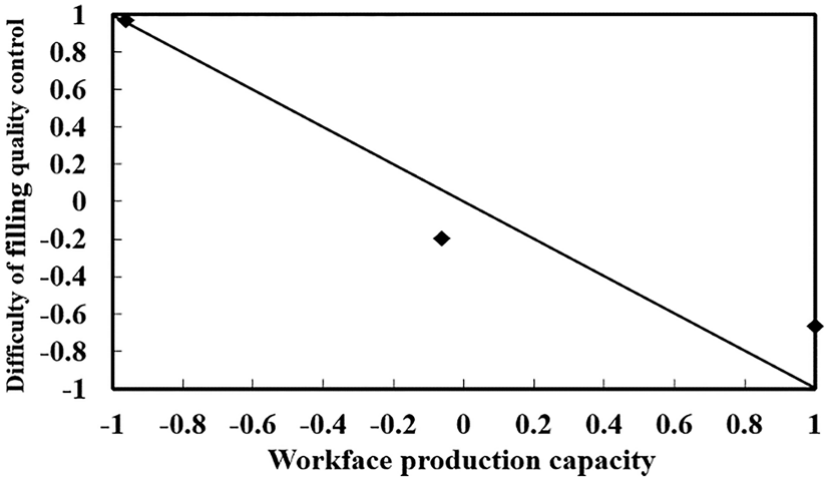
Correlation between working face production capacity and difficulty of filling quality control.

In fact, the correlation coefficient matrix ∏ has shown that there is a strong correlation between the evaluation indexes, as shown in Figs. 4 and 5, from which it can be seen that there is a significant positive correlation between the maintenance compensation fee and the degree of mining damage, and a significant negative correlation between the production capacity of the working face and the difficulty of quality control of the filling, which, if the correlation between the evaluation indexes is not taken into account, will greatly affect the accuracy of the evaluation results,. It proves the superiority of the Mahalanobis distance based on the correlation coefficient matrix compared with the traditional Euclidean distance in solving the bull's-eye distance.

Based on the above analysis, it can be concluded that the grey target model constructed in this paper integrates the advantages of the game theory combination to determine the optimal index weight, and uses the Mahalanobis distance based on the correlation coefficient matrix to solve the relative bullseye distance, taking into account the influence of the correlation relationship between the indicators. The evaluation results of the model are scientific and effective.

#### Test of model evaluation results

After specific analysis, Scheme 2, while ensuring low mining damage, high mining efficiency and low investment in filling, extracts more coal resources and achieves higher returns with lower costs. As a sub-optimal scheme, although Scheme 3 has more advantages than Scheme 2 in terms of lower investment in filling cost and more coal resources extracted, the extent of damage caused by mining is greater, adhering to the principle of protecting the mining area in a people-oriented manner, and considering that there is not a big difference in the recovery rate of coal resources in Scheme 2 compared with Scheme 3, Scheme 2, i.e., the general face-length, thickness-limiting, single-length-wall, backward-type, integrated, paste-filled coal mining, is considered as the optimal solution for this mining area. Therefore, it is determined that Scheme 2, i.e. general face long limit thickness single long wall backward integrated mining paste filling coal mining method, is the optimal mining plan for this mining area.The mining scheme is applied to the five mining areas of a coal mine, obtaining economic benefits of 189,531,600 yuan, and the impact degree of surface building mining is at the degree of very slight damage of class I, improving the economic benefits of coal mining and the safety of working face production. Through the on-site verification of the working face, it is proved that the improved grey target decision-making model is reasonable and feasible to be used in the selection of underpressure coal filling mining scheme after considering the above nine indexes.

In this paper, by constructing the game theory combination empowerment improved gray target decision-making model and constructing the evaluation index system for the preferred evaluation of the underpressure coal filling mining plan under the building, the application of the improved gray target model is carried out with the preferred evaluation of the filling mining plan of the five mining areas of a coal mine as an evaluation case, which verifies the validity of the present model. The specific application process of this model is shown as follows: (1) Decision makers need to first collect detailed data about the evaluation indexes constructed in this paper, which will be used as the basis of the model input; (2) Substitute the collected data into this model, and carry out calculations in accordance with the model operation steps listed in Sect. "Improved grey target decision-making model based on game-theoretic combinatorial empowerment" of this paper, and finally arrive at the evaluation results.

However, this model still faces some challenges: (1) the model is an evaluation method built on mathematical methods, are based on a certain theory and simplified to establish an evaluation model for coal mining, the mining situation of coal mines in different regions varies, so it is necessary to collect more information on the filling mining, strengthen the accuracy of the model's input data and the integrity of the analysis to improve the accuracy of the evaluation model; (2) the evaluation model constructed in this paper is more complex, so it requires more computing time, and there may be arithmetic errors in the computing process, it is recommended to program the model formulas to reduce the complexity and time cost of computing. The evaluation model constructed in this paper is more complex, so it needs more computing time, and there may be arithmetic errors in the arithmetic process, it is recommended to realize the arithmetic of the model formula through programming to reduce the arithmetic complexity and time cost.

## Conclusion

1. This paper focuses on the evaluation index system and decision-making model for the preferred selection of coal pressure filling mining scheme based on the set of solutions to be selected for coal pressure filling mining under buildings. The key indicators in the process of coal underfill mining are extracted from economy, technology and safety. Compared with other indicator systems in the field of coal underfill mining, the most important feature of the system is that it takes into account the economy of coal mining and the safety of coal underfill mining, and quantitatively evaluates the impact of the coal underfill mining scheme on the economic benefits of the enterprise and the stability of the ground surface.
2. An improved grey-target decision-making model based on game-theoretic combinatorial assignment is established, which combines the traditional grey-target decision-making model with game-theoretic combinatorial assignment method and Mars distance based on correlation coefficient matrix to construct the optimal weights of the indicators and take into account the correlation of the indicators so as to carry out the evaluation of the preferred selection of the coal mining plan under the building. The combination assignment method based on game theory takes into account the human subjective experience factor and the objective inherent properties of evaluation indexes, overcomes the limitations of the single subjective assignment method or objective assignment method, and determines the subjective and objective combination weights of the evaluation indexes more scientifically and reasonably, which increases the reasonableness of the evaluation results. The theory of positive and negative ideal solutions in the TOPSIS model is introduced into the traditional grey target decision-making model to construct positive and negative bull's-eye, which improves the defects of single bull's-eye that is easy to cause inaccurate evaluation results. At the same time, the Mahalanobis distance based on the correlation coefficient matrix is used to replace the European distance in the traditional grey target decision-making model, and the correlation between the evaluation indicators is fully considered to eliminate the influence of the correlation of the evaluation indicators on the accuracy of the evaluation results.
3. The results of a coal mine five mining area mining example show that: the game theory combination empowerment grey target decision-making model constructed in this paper can be used to serve for the preferred under-construction pressurised coal filling mining scheme, and its preferred mining scheme is consistent with the actual mining scheme of the working face, which provides a kind of theoretical basis for the decision-makers to evaluate the optimal scheme of the under-construction pressurised coal filling mining.

## Data Availability

Some or all data that support the findings of this study are available from the corresponding author upon reasonable request.
